# Protein salvador homolog 1 acts as a tumor suppressor and is modulated by hypermethylation in pancreatic ductal adenocarcinoma

**DOI:** 10.18632/oncotarget.17972

**Published:** 2017-05-18

**Authors:** Lei Wang, Mei Wang, Chenxi Hu, Pengping Li, Yun Qiao, Youyou Xia, Liang Liu, Xiaodong Jiang

**Affiliations:** ^1^ Department of Radiation Oncology, Lianyungang First People's Hospital, Jiangsu, People's Republic of China; ^2^ Tumor Laboratory, Department of Radiation Oncology, The Affiliated Lianyungang Hospital of Xuzhou Medical University, Jiangsu, People's Republic of China; ^3^ Department of Bioinformatics, School of Basic Medical Sciences, Nanjing Medical University, Nanjing, People's Republic of China; ^4^ Department of Radiation Oncology, The Affiliated Lianyungang Hospital of Xuzhou Medical University, Jiangsu, People's Republic of China

**Keywords:** SAV1, tumor suppressor, pancreatic ductal adenocarcinoma, hypermethylation

## Abstract

Salvador (SAV) is a gene product that contains two protein-protein interaction modules known as WW domains and is believed to act as a scaffolding protein for Hippo and Warts. SAV1 is the human homolog of Salvador, which is the most well characterized upstream signaling component of Hippo pathway. Although its role in some tumors is known, SAV1 function in other types of tumors, including pancreatic tumor, is still obscure. Here, we determined the role of SAV1 in pancreatic ductal adenocarcinoma (PDAC) development and progression. Our results revealed that SAV1 suppressed expression promoted PDAC invasion and migration, and repressed pancreatic cancer cells apoptosis. Moreover, SAV1 was silenced by hypermethylation. Thus, SAV1 worked as a cancer suppressor and it might be considered as a target for pancreatic cancer therapy.

## INTRODUCTION

The mammalian Hippo-Salvador signaling pathway has been implicated in the regulation of cell proliferation, cell death, tissue regeneration, and tumorigenesis [1–3]. Sav (SAV1 in mammals) is one of the most crucial members of the pathway that have been shown to restrict cell number by coordinating cell-cycle exit and apoptosis during Drosophila development [4]. In recent years, studies have been intensively focused on effects of the Hippo signaling in human tumorigenesis [5–8]. SAV1 is the human homolog of Salvador. SAV knockdown in mouse liver can induce hepatocellular carcinoma (HCC) development [9, 10]. Furthermore, SAV1 downregulation caused by 14q loss confers a survival and growth advantage on renal cell carcinoma (RCC) [11]. Chen et, al. demonstrated that Hippo signaling inactivation is correlated with poor overall survival in patient with pancreatic cancer [12]. However, despite all these evidences, the role of SAV1 in pancreatic ductal adenocarcinoma development is still unclear. Thus, further studies focusing on the role of SAV1 in the development and progression of some different types of tumors, including pancreatic tumor, are still needed.

Pancreatic cancer is the fourth leading cause of cancer-related death [13–15]. Pancreatic ductal adenocarcinoma (PDAC) comprises more than 85% of all pancreatic cancer and has extremely poor prognosis, with an overall five-year survival rate at less than 5% [14, 16]. Thus, the identification of the molecular mechanisms characterizing pancreatic cancer development and progression is urgently needed [14].

In the present study, we aimed to determine SAV1 role and mechanism in pancreatic cancer development and progression. Our results showed that SAV1 suppressed expression in pancreatic cancer was due to hypermethylation. Furthermore, SAV1 decreased pancreatic cancer cells invasion and migration, and could promoted pancreatic cancer cells apoptosis, providing new insights into its role in this type of tumor.

## RESULTS

### SAV1 expression in pancreatic cancer tissues and cell lines

**Figure 1:**
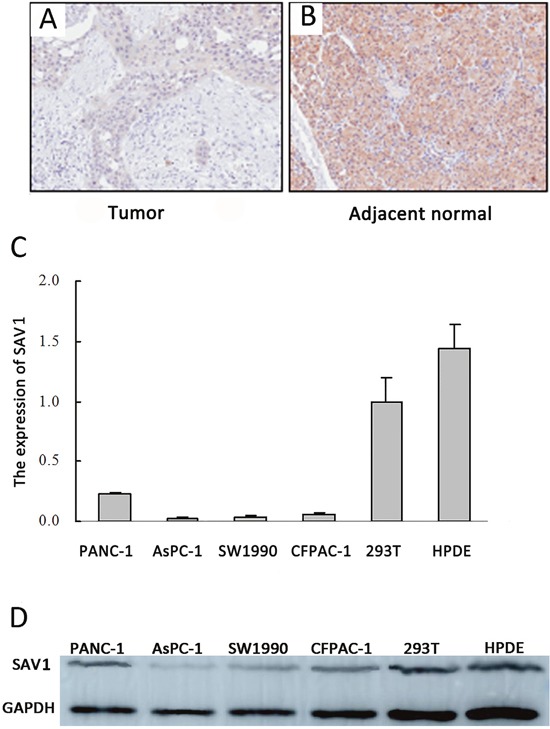
Expression of SAV1 in pancreatic cancer tissues and cell lines Immunostaining of SAV1 in pancreatic tumor tissue microarray (TMA) with a specific anti-SAV1 antibody. **(A)** Tumor tissues showing weak SAV1 staining. **(B)** Adjacent pancreatic tissues showing strong SAV1 staining. **(C)** Histograms of SAV1 mRNA level in six various types of cells, including four pancreatic cancer cell lines (PANC-1, AsPC-1, CFPAC-1, SW1990) and the nontumorigenic cell lines (293Tand HPDE). **(D)** Verification of expression of SAV1 in pancreatic cancer cell lines and nontumorigenic cell lines by western blot.

To investigate SAV1 role on pancreatic cancer development and progression, immunohistochemical analysis of tissue microarray was performed to analyze SAV1 expression in the TMA which contained 83 primary pancreatic tumor tissues and 83 tumor adjacent tissues. Our results showed a strong SAV1 staining localized predominantly in the cytoplasm of most of the adjacent normal tissue, whereas SAV1 was not expressed in pancreatic cancer tissues (Figure 1A). SAV1 expression in nonmalignant tissue was much higher than in cancer tissue (Figure 1B). We then further investigated the relationship between SAV1 expression and pancreatic tumor clinicopathological parameters. The results showed that SAV1 expression was not significantly associated with gender, age, tumor size, tumor location, nerve invasion, T stages, M stages and TNM stages of pancreatic cancer (*P*>0.05, Table 1), but negatively correlated with N stages (*P=0.009*) and differentiation (*P*=0.025). SAV1 expression was also evaluated in four PDAC cell lines and one normal human pancreatic duct epithelial cell line (HPDE). As shown in Figure 1C and 1D, SAV1 mRNA and protein levels were lower in the four PDAC cell lines compared to their levels in HPDE. Thus, these results indicated that SAV1 was low expressed in PDAC suggesting a role as tumor suppressor in PDAC progression.

**Table 1:** Relationship between SAV1 expression level and clinicopathologic variables in 83 PDAC tissues

Clinicopathologic parameters	Cases (n=83)	SAV1 immunostaining		*P* value
		Weak positive	Strong positive	
Age(years)				
<62	37	18	19	1.000
≥62	46	23	23	
Gender				
Male	53	30	23	0.129
Female	30	11	19	
Tumour size(cm)				
≤4	47	22	25	0.751
>4	36	19	17	
Nervous invasion				
Negative	49	27	22	0.306
Positive	34	14	20	
Differentiation				
Poor	24	17	7	0.025*
Well	59	24	35	
Tumor location				
Head, neck	49	23	26	0.753
Body, tail	34	18	16	
T classification				
T1+T2	70	36	34	0.548
T3+T4	13	5	8	
N classification				
No	53	20	33	0.009*
N1	30	21	9	
M classification				
M0	82	41	41	1.000
M1	1	0	1	
TNM stage				
I-II	82	41	41	1.000
III-IV	1	0	1	

### Promoter hypermethylation silenced SAV1 in pancreatic cancer

**Figure 2:**
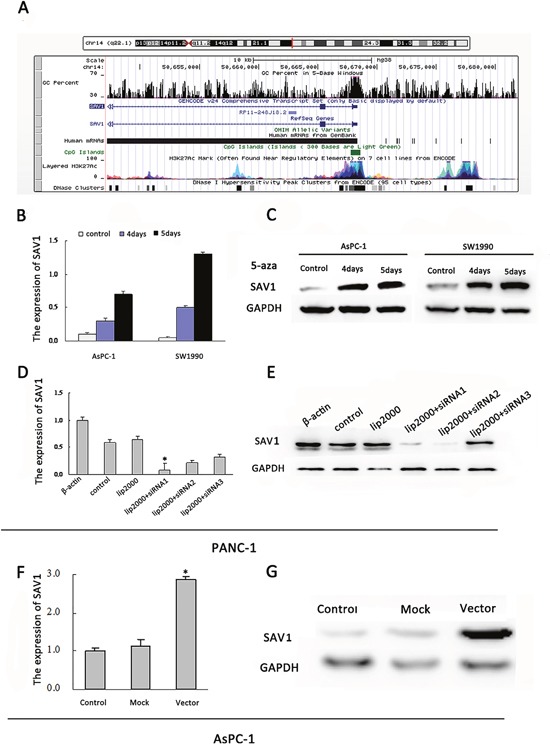
Regulation of SAV1 expression by promoter hypermethylation in pancreatic cancer and validation of transfection **(A)** Diagram of CpG islands in the promoter region of SAV1. Reactivation of SAV1 expression. AsPC-1and SW1990 cells were treated with 5-azaC (5μM) for 4 and 5 days. Quantitative real-time PCR **(B)** and western blot **(C)** were used to analyze the mRNA and protein levels of SAV1, respectively. **(D)** Quantitative real-time PCRanalyses of SAV1 expression in PANC-1 cells underwent transient transfection of SAV1 siRNA (siRNA1, siRNA2, and siRNA3). **(E)** SAV1 protein expression in PANC-1 cells transfected with SAV1 siRNA (siRNA1, siRNA2, and siRNA3) was confirmed by western blot analysis. **(F)** PCR analyses of SAV1 expression in AsPC-1 cells underwent stably transfection. **(G)** SAV1 protein expression in AsPC-1 cells transfected with SAV1-constructed plasmid.

SAV1 gene promoter region contains typical CpG islands (Figure 2A). However, the effects of SAV1 promoter hypermethylation on pancreatic cancer have not been demonstrated. We incubated AsPC-1 and SW1990 cells, two PDAC cell lines not expressing SAV1, in fresh medium with or without the demethylating agent 5-azaC. SAV1 mRNA and protein expression were restored in the 5-azaC treated groups (Figure 2B and 2C). These results demonstrated that promoter hypermethylation led to SAV1 silencing in pancreatic cancer.

### SAV1 suppressed pancreatic cancer cells migration and invasion

**Figure 3:**
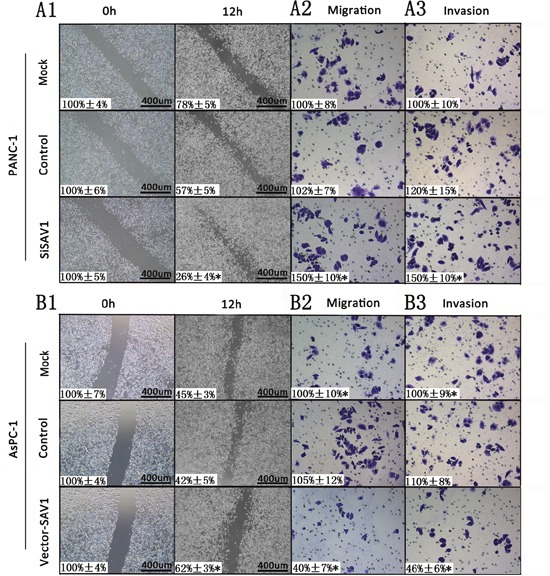
Influence of SAV1 expression on migration and invasion of pancreatic cancer cells For cell scratch wound assay, PANC-1 **(A1)** and AsPC-1 cells **(B1)** were transfected with siSAV1 and pLenti-EF1a-EGFP-P2A-Puro-CMV-SAV1-3Flag for 48 hours, resprectively. The cultures were then wounded by scratching and maintained for additional 12 hours. Cell cultures were photographed and migration was measured by the cell-free areas in multiple fields (inserted number represented the percentage of gap areas±SD). The migration and invasion of PANC-1 (**A2** and **A3**) and AsPC-1 (**B2** and **B3**) cells were analyzed by the cell migration and invasion assays as described in the Materials and Methods. Representative cancer cell migrated or invaded were photographed, data represent mean±SD of triplicates. Note: *, *P*<0.05 (one way ANOVA) in a comparison of the siSAV1 or vector-SAV1treated groups with the mock and control groups.

Since the role of SAV1 in pancreatic cancer is still unclear, to further understand its role we silenced or overexpressed SAV1 and analyzed the effect in pancreatic cancer cells. Both siRNA#1 and siRNA#2 could silence SAV1, but siRNA#1 was more effective (Figure 2D and 2E). Thus, siRNA#1 (siSAV1) was used for our further experiments. We then analyze the effect of SAV1 on pancreatic cancer growth and metastasis. We ectopically expressed SAV1 in AsPC-1 cells, and silenced SAV1 in PANC-1 cells, another PDAC cell line. To assess the impact of SAV1 expression on pancreatic cancer migration and invasion, the transfected PANC-1 cells were wounded by scratching and the results showed that the silencing of SAV1 promoted PANC-1 cells flattening and spreading (Figure 3A1, *, *P*<0.05), while ectopic expression of SAV1 in AsPC-1 cells wounded by scratching resulted in a decreased cell flattening and spreading (Figure 3B1, *P<0.05). Furthermore, the results of migration and invasion assay revealed that SAV1 silencing promoted PANC-1 cells migration and invasion (Figure 3A2 and 3A3, *P<0.05), whereas SAV1 overexpression repressed AsPC-1 cells migration and invasion (Figure 3B2 and 3B3, *, *P*<0.05).

### SAV1 induced pancreatic cancer cells apoptosis

**Figure 4:**
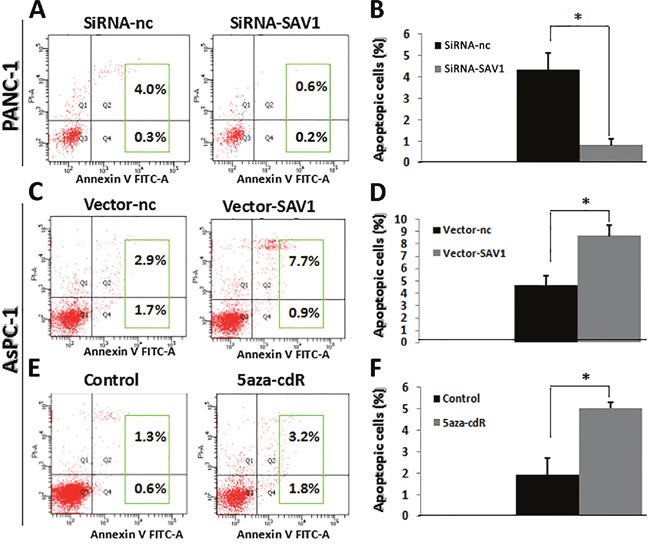
Influence of SAV1 expression on apoptosis of pancreatic cancer cells **(A)** and **(B)** Knockdown of SAV1 repressed the apoptosis of PANC-1 cells. **(C)** and **(D)** Overexpression of SAV1 promoted the apoptosis of AsPC-1 cells. **(E)** and **(F)** AsPC-1cells treated with the demethylating agent 5-azaC resulted in restored expression of SAV1. Furthermore, restored SAV1 promoted the apoptosis of AsPC-1 cells.

Cell apoptosis was examined by flow cytometry and results showed that the percentage of apoptotic cells was significantly higher in PANC-1/siRNA-nc group than in PANC-1/siRNA-SAV1 (Figure 4A and 4B,*P<0.05) and the percentage of apoptotic cells was significantly higher in AsPC-1/SAV1-vector group than in AsPC-1/nc-vector (Figure 4C and 4D,*, *P*<0.05). SAV1 silencing repressed apoptosis of PANC-1 cells, whereas SAV1 overexpression promoted apoptosis of AsPC-1 cells.

In addition, the percentage of apoptotic cells was significantly higher in 5-azaC treated AsPC-1 cells than that in the AsPC-1 cells without 5-azaC in the medium (negative control) (Figure 4E and 4F, *P<0.05).

## DISCUSSION

SAV1 acts as a tumor suppressor in tumors other than RCC. However, SAV1 expression mechanism and effect have not been fully evaluated in pancreatic cancer. In the current study, we demonstrated that SAV1 suppressed expression resulted in a promotion of pancreatic cancer development and its suppression was due to promoter hypermethylation. Researches have demonstrated that polycomb group (PcG) proteins interacted with DNA methyltransferases (DNMTs) and led to DNA methylation of certain gene promoters [17, 18]. PcG of proteins contain two multimeric protein complexes, that are, polycomb complex 1 (PRC1) and polycomb complex 2 (PRC2) [19]. Our results, together with these evidences suggested that decreased expression of SAV1 in pancreatic cancer was due to promoter hypermethylation.

SAV1 on pancreatic cancer metastasis and apoptosis were also assessed. Our findings indicated that SAV1 silencing promoted migration and invasion, repressed apoptosis;whereas SAV1 overexpression repressed migration and invasion, promoted apoptosis.

In conclusion, this study identified for the first time the suppressor role of SAV1 in pancreatic cancer development and progression. Overall, these findings not only demonstrated the molecular mechanism characterizing pancreatic cancer development and progression, but also indicated that demethylating agents might be a promising new strategy for pancreatic cancer therapy, which required further studies.

## MATERIALS AND METHODS

### Cell lines and culture conditions

Four human pancreatic cancer cell lines PANC-1, SW1990, AsPC-1, CFPAC-1 cells and one normal human pancreatic duct epithelial cell line (HPDE) were purchased from the American Type Culture Collection (ATCC). All these cell lines were cultured in RPMI-1640 medium (Gibco BRL, San Francisco, CA, USA) containing 100 U/ml penicillin, 100ug/ml streptomycin and 10% fetal bovine serum (Invitrogen, Carlsbad, CA, USA) at 37°C in a 5% CO2-humidified incubator.

### Human tissue specimens and immunohistochemical analysis

Tissue microarray and immunohistochemical analysis was performed as previously described [20]. Tissues from 83 patients with pancreatic ductal adenocacinoma were obtained from the Pancreatic Cancer Tissue Bank at Shanghai First People's Hospital (Shanghai, PR China). The 83 primary tumors samples were associated to 83 tissues samples from adjacent healthy tissue. Tissue microarray was prepared and processed for immunostaining using anti-SAV1(1:200 dilutions;no.ab105105, AbcamInc., USA) antibodies. The staining results were scored by two investigators blinded to the clinical data as previously described [21].

### RNA interference (RNAi) and transfection

Small interfering RNAs (siRNAs) to silence SAV1 were designed and synthesized by Genechem (Ribobio Co. Ltd., Guangzhou, China). Negative control siRNA (Invitrogen, Grand Island, NY, US) was used as control. siRNAs transfection into pancreatic cancer cells was performed using Lipofectamine 2000 CD (Invitrogen) transfection reagent. For transient transfection, cells were transfected with siRNA at different doses as indicated for 48 hours before the performance of functional assays. Pancreatic cancer cells treated with transfection reagent alone were used as mock controls.

Lentivirus and negative control were designed and synthesized by Genechem (Obio Technology Co. Ltd., Shanghai, China). The corresponding vector was pLenti-EF1a-EGFP-P2A-Puro-CMV-SAV1-3Flag. PANC-1 and CFPAC-1 cells were cultured in six-well plates and transfected with Lentivirus and negative control following the manufacturer's protocol. Cells were treated with puromycin (1 ug/mL) (InvivoGen, San Diego, CA, USA) to produce stable transfected cells (AsPC-1/nc-vector, AsPC-1/SAV1-vector,) for further experiments. SAV1 silencing and overexpression were confirmed by Western blot and Real-time-quantitative polymerase chain reaction (RT-qPCR) at 48 hours post-transfection.

### Treatment with 5-aza-2′-deoxycytidine (5-azaC)

Pancreatic cancer cell lines(AsPC-1, SW1990) were treated with 5-azaC at 5 μM for 4 and 5 days and total RNA and proteins were extracted and further analyzed by RT-qPCR and Western blot.

### RT-qPCR

RT-qPCR analysis to evaluate SAV1 expression was performed using total RNA and the SYBR green reagent with an ABI Prism 7000HT sequence detection system [22]. The sequences of the PCR primers were as follows: SAV1, 5′- GCAGGGGAAGTACGTGAAGA-3′ (forward) and 5′- GCATTAGGGCTTGAATCTGG-3′ (reverse); and GAPDH, 5′- CCCCGCTACTCCTCCTCCTAAG-3′ (forward) and 5′-TCCACGACCAGTTGTCC ATTCC-3′ (reverse).

### Western blot analysis

Standard Western blotting was carried out using primary antibodies against SAV1 (Abcam, USA, 1:500 dilution) and GAPDH (Sigma, USA, 1:10000 dilution). Goat anti-Rabbit IgG (Cst, USA, 1:5000 dilution) was used as secondary antibody.

### *In vitro* scratch-wound healing assay

SAV1 siRNA was transfected in PANC-1 cells and lentivirus was transfected in AsPC-1 cells. In brief, when cells reached 90-95% confluence in 6-well plates, the wound was generated by scratching the cell monolayer with a 10 μL pipette tip. Cells were washed with serum-free DMEM and photographs were taken at 0 and 12 hours. The *in vitro* wound filling was determined by measuring the cell-free areas in multiple fields using a service provided by Wimasis, which allows users to upload their images on line, then it performs their analysis and the results can be downloaded to the researcher's server [23].

### Tumor cell invasion/migration assay

PANC-1 and AsPC-1 cells were transfected for 12 hours with different reagents corresponding to different groups (mock, control, siSAV1 or mock, control, vector-SAV1). Cells in each group were trypsinized and 2×10^4^ to 5×10^4^ cells in a 300 μL volume of serum-free medium were placed in the upper parts of modified Boyden chambers with a Matrigel-coated/uncoated membrane (Millipore). For both cell lines, 500 μL of DMEM with 10% fetal bovine serum was used as chemoattractant and added to the lower chamber. After 24 hours of incubation, invasive/migrated cells were fixed, stained and counted under a microscope in five randomly selected fields at a magnification of 200×. Migrated cells were photographed using Nikon Digital Sight DS-U2 (Nikon, Tokyo, Japan) and Olympus BX50 microscopes (Olympus Optical Co. Ltd., Tokyo, Japan).

### Cell apoptosis analysis by flow cytometry

AsPC-1 and PANC-1 were collected and fixed in 70% ice-cold ethanol at 4°C overnight. After washing with PBS, cells were incubated with 100 μg/ml RNase A at 37°C for 20 min. Cells were subsequently stained with propidium iodide (50μg/mL). To assess apoptosis, unfixed tumour cells were washed with PBS and incubated with Annexin V and propidium iodide (BD Biosciences, San Jose, CA, USA) according to the manufacturer's instruction.

### Statistical analysis

SPSS software program (version 16.0; SPSS Inc, New York, US) was used for statistical analysis. Experiments were repeated at least three times and results are shown as mean±standard deviation (s.d.). Two-tailed χ^2^ test or Fisher's exact test was used to determine the significance of the difference among the covariates of patient specimens. Data significance was determined using Student *t*-test (two-tailed) or one way ANOVA. *P* values less than 0.05 were considered statistically significant.
